# Rare Earth Complexes of Europium(II) and Substituted Bis(pyrazolyl)borates with High Photoluminescence Efficiency

**DOI:** 10.3390/molecules27228053

**Published:** 2022-11-20

**Authors:** Ruoyao Guo, Zifeng Zhao, Aoben Wu, Yuqin Li, Kezhi Wang, Zuqiang Bian, Zhiwei Liu

**Affiliations:** 1 Beijing National Laboratory for Molecular Sciences, State Key Laboratory of Rare Earth Materials Chemistry and Applications, College of Chemistry and Molecular Engineering, Peking University, Beijing 100871, China; 2 Beijing Key Laboratory of Energy Conversion and Storage Materials, College of Chemistry, Beijing Normal University, Beijing 100875, China

**Keywords:** europium(II) complex, d-f transition, photoluminescence, bis(pyrazolyl)borates

## Abstract

Rare earth europium(II) complexes based on d-f transition luminescence have characteristics of broad emission spectra, tunable emission colors and short excited state lifetimes, showing great potential in display, lighting and other fields. In this work, four complexes of Eu(II) and bis(pyrazolyl)borate ligands, where pyrazolyl stands for pyrazolyl, 3-methylpyrazolyl, 3,5-dimethylpyrazolyl or 3-trifluoromethylpyrazole, were designed and synthesized. Due to the varied steric hindrance of the ligands, different numbers of solvent molecules (tetrahydrofuran) are participated to saturate the coordination structure. These complexes showed blue-green to yellow emissions with maximum wavelength in the range of 490–560 nm, and short excited state lifetimes of 30–540 ns. Among them, the highest photoluminescence quantum yield can reach 100%. In addition, when the complexes were heated under vacuum or nitrogen atmosphere, they finally transformed into the complexes of Eu(II) and corresponding tri(pyrazolyl)borate ligands and sublimated away.

## 1. Introduction

Rare earth complexes are widely studied as luminescent materials in many fields because of their rich orbital energy levels and unique luminescent properties [1,2], such as organic light emitting diodes (OLEDs) [3,4,5,6,7], bio-imaging [8,9,10,11,12], light conversion and anti-counterfeiting [12,13]. The luminescence mechanism of rare earth complexes can be mainly divided into two types: f-f transition and d-f transition. So far, the former has been more widely studied. The 4f orbitals are in the inner shell and shielded by the filled 5s^2^5p^6^ sub-shells, resulting in the relatively fixed emission wavelengths and narrow-line emission spectra. However, this also leads to the limitation of the luminescent colors to a certain degree [14]. In addition, the excited state lifetimes of parity forbidden f-f transitions are long (usually on the order of a millisecond) [6]. Different from f-f transitions, the luminescent colors of d-f transitions are adjustable from ultraviolet to infrared by changing the chemical and/or electronic structure of coordination ligands because the 5d orbitals are easily affected by coordination environment [15,16,17,18,19,20]. Moreover, parity allowed d-f transitions cause shorter excited state lifetimes (usually on the order of nanosecond to microsecond) [15,16,20,21,22,23,24]. These unique properties make the d-f transition-based rare earth complexes have great potential in many fields.

**Figure 1:**
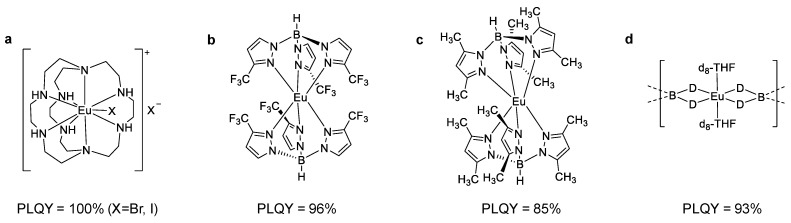
The previously reported Eu(II) complexes with high PLQYs.

Eu(II) complexes can exhibit luminescence based on d-f transition. The 5d-4f transition energy of free Eu(II) ion is about 3.73 eV [25]. Therefore, Eu(II) complexes can realize emissions from ultraviolet to infrared. The reported ligands coordinated with Eu(II) mainly include crown ethers [26], cryptands [15,27,28,29,30,31,32,33,34], tri(pyrazolyl)borates [16,20,35,36,37], cyclopentadienyls [38,39,40,41] and other kinds of ligands [42,43]. Among them, the photoluminescence quantum yield (PLQY) of the complex formed by Eu(II) and 1,4,7,10,13,16,21,24-octaazabicyclo[8.8.8]hexacosane reached 100% (Figure 1a), which is the only example [15]. Eu(II) complexes formed with hydrotris(3-trifluoromethylpyrazolyl)borate and hydrotris(3,5-dimethylpyrazolyl)borate also have high PLQYs, i.e., 96% (Figure 1b) and 85% (Figure 1c), respectively [16,35], and the PLQY of Eu(II) complex with deuterated BD_4_^-^ and tetrahydrofuran can reach 93% (Figure 1d). While the PLQYs of other Eu(II) complexes ranged from 3% to 75%. Different from aforementioned ligands, we propose that bis(pyrazolyl)borates (Bp) have similar properties to tri(pyrazolyl)borates (Tp), such as small conjugate structure, high energy level and rigid structure and may also coordinate well with Eu(II). To the best of our knowledge, no luminescent Eu(II) complex with Bp type ligand has been studied. Herein four complexes of Eu(II) and Bp ligands with different substituents at pyrazole’s three-site and five-site, i.e., pyrazolyl (Eu-Bp), 3-methylpyrazolyl (Eu-Bp^Me^), 3,5-dimethylpyrazolyl (Eu-Bp^Me2^) and 3-trifluoromethylpyrazole (Eu-Bp^CF3^) were designed and synthesized, and their structures, photophysical properties and thermal stabilities were studied systematically. Two complexes achieved high photoluminescence efficiency with PLQYs up to 100%. In addition, when heated under vacuum or nitrogen atmosphere, these complexes can finally transform into complexes of Eu(II) and corresponding Tp ligands.

## 2. Results and Discussion

### 2.1. Synthesis and Structures

**Scheme 1:**
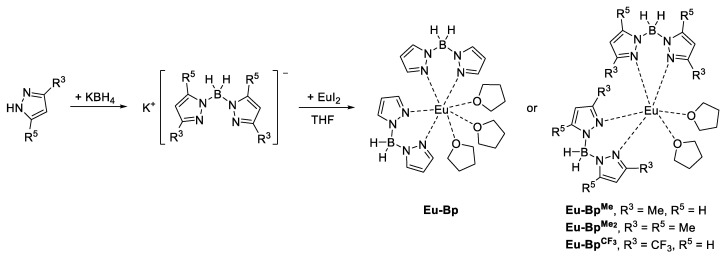
The chemical structures and synthetic routes of the investigated Eu(II) complexes.

The chemical structures and synthetic routes of the four Eu(II) complexes are shown in Scheme 1. In theory, the negative univalent ligands and positive divalent Eu(II) ions can form neutral complexes in the ratio of 2:1. The Bp ligands were synthesized by pyrazole with different substituents and potassium borohydride. They were mixed in the ratio of 2.2:1 and heated until two equivalent hydrogen was released to obtain the corresponding potassium dihydrobis(pyrazolyl)borate [44,45,46]. Then, the potassium salts and europium(II) iodide were mixed and stirred in tetrahydrofuran with a ratio of 2:1, and the Eu(II) complexes were purified by recrystallization.

**Figure 2:**
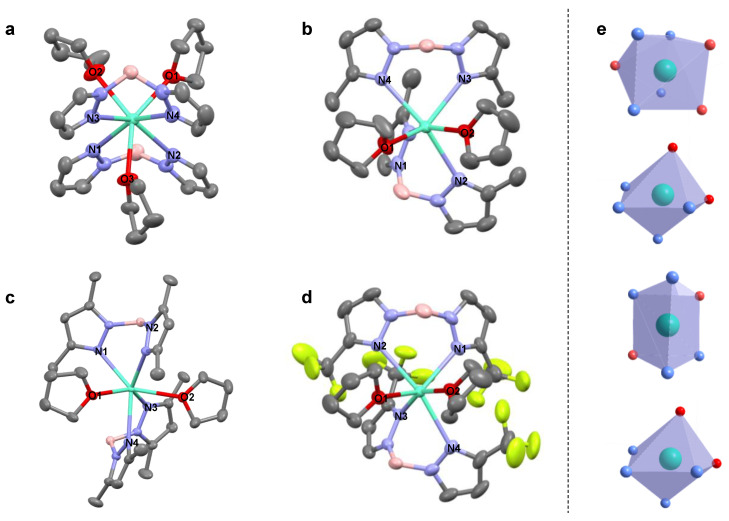
The crystal structures of the investigated Eu(II) complexes: (**a**) Eu-Bp; (**b**) Eu-Bp^Me^; (**c**) Eu-Bp^Me2^; (**d**) Eu-Bp^CF3^; and (**e**) coordination geometry of the four Eu(II) complexes, Eu-Bp, Eu-Bp^Me^, Eu-Bp^Me2^ and Eu-Bp^CF3^ are arranged from top to bottom. For clarity, the hydrogen atoms are omitted. Eu atoms are represented in cyan, O in red, B in pink, N in blue, C in gray, and F in green.

The single crystals of Eu-Bp, Eu-Bp^Me^, Eu-Bp^Me2^ and Eu-Bp^CF3^ were obtained by slowly evaporating the mixed solvent of tetrahydrofuran and n-hexane. Their crystal structures were determined by X-ray diffraction, as shown in Figure 2. The coordination bond lengths of central Eu(II) and coordination atoms (N, O) are shown in Table 1. Other parameters are shown in Table S1.

The four complexes have two different coordination models owing to the varied steric hindrance of the Bp ligands. In the crystal structure of Eu-Bp, the central Eu(II) not only coordinates with two ligands, but also coordinates with three solvent molecules (tetrahydrofuran) to achieve a saturated structure, due to the small steric hindrance of dihydrobis(pyrazolyl)borate. The coordination polyhedron formed by four N atoms and three O atoms can be regarded as an irregular triangular prism capped on one side surface. The average bond length of Eu-N is 2.652 Å, and the average bond length of Eu-O is 2.629 Å (Table 1).

In the crystal structures of Eu-Bp^Me^, Eu-Bp^Me2^ and Eu-Bp^CF3^, the steric hindrance of the Bp ligands increases due to introducing methyl or trifluoromethyl on the three-site of the pyrazolyl rings. Therefore, the central Eu(II) coordinates with two Bp ligands and only two solvent molecules (tetrahydrofuran). The coordination polyhedron formed by four N atoms and two O atoms can be regarded as a deformed octahedron (Eu-Bp^Me^ and Eu-Bp^CF3^) or a triangular prism (Eu-Bp^Me2^). The average bond lengths of Eu-N are 2.634 Å, 2.640 Å and 2.692 Å, and the average bond lengths of Eu-O are 2.572 Å, 2.579 Å and 2.558 Å in Eu-Bp^Me^, Eu-Bp^Me2^ and Eu-Bp^CF3^, respectively (Table 1). Comparing Eu-Bp^Me^ and Eu-Bp^CF3^, it is found that the increase of steric hindrance as well as electron-withdrawing property of the ligands weakens Eu-N bonds, thus lengthening Eu-N bonds. But at the same time, the smaller tetrahydrofuran molecules can be closer to the central Eu(II), which shortens the bond lengths of Eu-O. Comparing Eu-Bp^Me^ and Eu-Bp^Me2^, it shows that the introduction of methyl on the five-site of the pyrazolyl rings has little effect on steric hindrance of the ligands. The bond lengths of Eu-N and Eu-O of Eu-Bp^Me2^ are slightly longer than Eu-Bp^Me^.

### 2.2. Photophysical Properties

The UV-Vis absorption spectra of the four Eu(II) complexes in tetrahydrofuran solution (1 × 10^−3^ M) were measured, showing in Figure S1. Their main absorption peaks and corresponding molar absorption coefficients (ϵ) are shown in Table S2. Eu-Bp shows absorption peaks at around 265 nm (ϵ ~ 1123 L mol^−1^ cm^−1^) and 353 nm (ϵ ~ 882 L mol^−1^ cm^−1^). Eu-Bp^Me^ shows absorption peaks at around 259 nm (ϵ ~ 897 L mol^−1^ cm^−1^) and 360 nm (ϵ ~ 806 L mol^−1^ cm^−1^). Eu-Bp^Me2^ shows absorption peaks at around 256 nm (ϵ ~ 966 L mol^−1^ cm^−1^), 336 nm (ϵ ~ 1008 L mol^−1^ cm^−1^) and 354 nm (ϵ ~ 1000 L mol^−1^ cm^−1^), and Eu-Bp^CF3^ shows absorption peaks at 252 nm (ϵ ~ 1365 L mol^−1^ cm^−1^), 303 nm (ϵ ~ 1397 L mol^−1^ cm^−1^) and 346 nm (ϵ ~ 1134 L mol^−1^ cm^−1^). These absorption bands can be attributed to the Laporte- and spin-allowed 4f–5d transitions of Eu(II), for their absorbance intensity is consistent with reported Eu(II) complexes [15,16,34,43].

**Figure 3:**
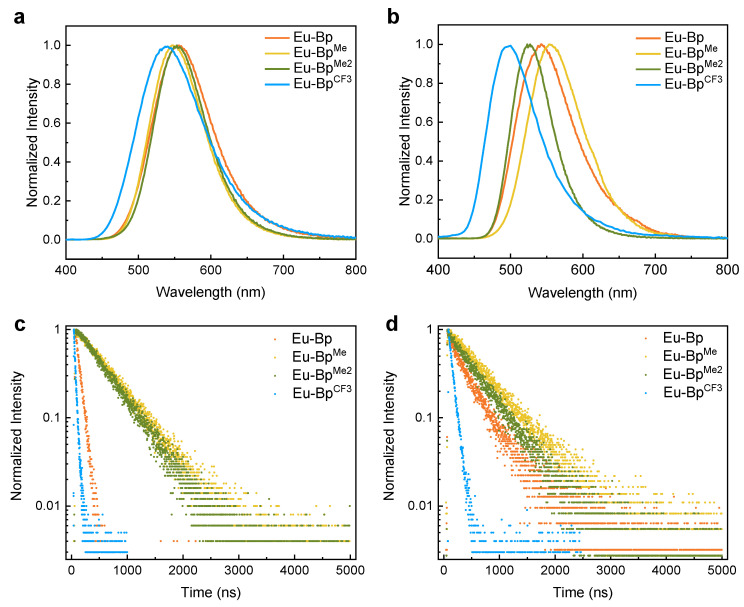
The photophysical properties of the four Eu(II) complexes: (**a**) emission spectra in tetrahydrofuran solution (1 × 10^−3^ M). The excitation wavelengths are all 365 nm. (**b**) Emission spectra as solid powder. The excitation wavelengths are 370 nm, 380 nm, 380 nm and 300 nm for Eu-Bp, Eu-Bp^Me^, Eu-Bp^Me2^ and Eu-Bp^CF3^, respectively. (**c**) Transient photoluminescence decay curves in tetrahydrofuran solution (1 × 10^−3^ M). The excitation wavelengths are all 365 nm, and the emission wavelengths are 550 nm, 550 nm, 550 nm and 540 nm for Eu-Bp, Eu-Bp^Me^, Eu-Bp^Me2^ and Eu-Bp^CF3^, respectively. (**d**) Transient photoluminescence decay curves as solid powder. The excitation wavelengths are 405 nm, 405 nm, 405 nm and 320 nm, respectively, and the emission wavelengths are 545 nm, 555 nm, 525 nm and 500 nm for Eu-Bp, Eu-Bp^Me^, Eu-Bp^Me2^ and Eu-Bp^CF3^, respectively.

The excitation and emission spectra of the four Eu(II) complexes in tetrahydrofuran solution (1 × 10^−3^ M) are shown in Figure S2a and Figure 3a. The excitation bands are basically the same as their absorption bands. The main excitation peaks are shown in Table S2. The maximum emission peaks (λ_em_) of Eu-Bp, Eu-Bp^Me^, Eu-Bp^Me2^ and Eu-Bp^CF3^ are located at 556 nm, 546 nm, 553 nm and 541 nm, respectively (Table 2). Their emission peaks are relatively close, which may be due to the influence of the solvent. Their emission colors can be represented by Commission Internationale de l’Eclairage (CIE) chromaticity coordinates (Figure S3a, Table 2), showing as yellow-green. All the four complexes show broadband emissions, and their full widths at half maximum (FWHMs) are 96 nm, 88 nm, 83 nm and 109 nm, respectively (Table 2).

The excitation and emission spectra of the four Eu(II) complexes as solid powder are shown in Figure S2b and Figure 3b. The excitation bands are also basically the same as their absorption bands. The λ_em_ of Eu-Bp, Eu-Bp^Me^, Eu-Bp^Me2^ and Eu-Bp^CF3^ are located at 542 nm, 554 nm 526 nm and 497 nm (Table 2), demonstrating emission colors as yellow-green, yellow, green and blue-green, respectively. The CIE 1931 chromaticity diagram of these complexes (Figure S3b and Table 2) can visually represent their emission colors. Except Eu-Bp with different coordination model, the λ_em_ of Eu-Bp^Me^, Eu-Bp^Me2^ and Eu-Bp^CF3^ are blue shifted gradually. This is because the emission energy of an Eu(II) complex is generally related to the average bond lengths [47]. The coordination ability of Bp ligands is much stronger than that of tetrahydrofuran, so the change of Eu-N bond lengths is the main influencing factor. A longer bond length makes a weaker ligand field splitting of 5d orbital, resulting in higher energy level of the lowest energy 5d orbital. The transition energy from the lowest energy 5d orbital to 4f orbital is higher as well, assuming that the energy of 4f orbital is unchanged. Therefore, with the increase of the average Eu-N bond lengths of Eu-Bp^Me^, Eu-Bp^Me2^ and Eu-Bp^CF3^, their maximum emission peaks are blue shifted. Considering a much rigid environment in solid powder, the FWHMs are narrower than those in solution, which are 94 nm, 86 nm, 66 nm and 81 nm for Eu-Bp, Eu-Bp^Me^, Eu-Bp^Me2^ and Eu-Bp^CF3^, respectively (Table 2).

The transient photoluminescence decay curves of the four Eu(II) complexes in tetrahydrofuran solution (1 × 10^−3^ M) and as solid powder are shown in Figure 3c,d, respectively. The fitting results of their excited state lifetimes (τ) are 78 ns, 530 ns, 475 ns and 31 ns in tetrahydrofuran solution (1 × 10^−3^ M), and 379 ns, 543 ns, 488 ns and 153 ns as solid powder, respectively (Table 2). These lifetimes are comparable to those of the reported Eu(II) complexes [15,16,20]. The PLQYs of the four Eu(II) complexes are 11%, 76%, 76% and 5% in tetrahydrofuran solution (1 × 10^−3^ M), and 37%, 100%, 100% and 20% as solid powder, respectively (Table 2). The PLQYs of Eu-Bp^Me^ and Eu-Bp^Me2^ are the highest among the reported Eu(II) complexes [20]. To further understand the photophysical properties of these complexes, their radiative rate constants (*k*_r_) and non-radiative rate constants (*k*_nr_) both in solution and in solid powder were deduced from their PLQYs and excited state lifetimes, showing in Table 2. There is little difference in their radiative rate constants; thus, the difference in their PLQYs can mainly be attributed to the obvious change in non-radiative rate constants. For these complexes, the smaller steric hindrance of the ligands (e.g., Eu-Bp) or the longer distance between the ligands and central Eu(II) (e.g., Eu-Bp^CF3^) may make their luminescence easier to be quenched, thus increasing the non-radiative rate constants and decreasing the PLQYs. While in solution, the non-radiative rate constants are higher, and the PLQYs are lower to a certain degree compared with solid powder.

### 2.3. DFT Calculations

The UV-Vis absorption spectra and hole-electron analysis of the four Eu(II) complexes were calculated with time-dependent density functional theory (TD-DFT). The results of the absorption spectra are shown in Figure S4 and Table S3. The calculated maximum molar extinction coefficients are in the range of 800 L mol^−1^ cm^−1^ to 1400 L mol^−1^ cm^−1^, which are close to our experimental results and consistent with the previously reported 4f-5d transitions of Eu(II) complexes [15,16,34,43]. The calculated energies of the lowest excited states for Eu-Bp, Eu-Bp^Me^, Eu-Bp^Me2^ and Eu-Bp^CF3^ are 2.97 eV (418 nm), 2.87 eV (432 nm), 2.95 eV (420 nm) and 3.12 eV (398 nm), respectively. The trend is consistent with the experimental result. The hole-electron analyses of the lowest excited states are shown in Figure S5–S8. The hole is completely contributed by the excitation of 4f orbitals, while the electron is mainly contributed by 5d orbitals. Therefore, it can be considered that the luminescence of these four complexes comes from 5d-4f transitions.

### 2.4. Thermal Stability

The four Eu(II) complexes Eu-Bp, Eu-Bp^Me^, Eu-Bp^Me2^, and Eu-Bp^CF3^ were heated under the pressure of 10^−4^ Pa to test if they are sublimable. The colors and luminescent colors of the solid powders obtained by sublimation had changed significantly compared with the powders before sublimation. It is confirmed by elemental analyses that the solid powders obtained by sublimation are complexes of Eu(II) and the corresponding tri(pyrazolyl)borate ligands, i.e., Eu-Tp, Eu-Tp^Me^, Eu-Tp^Me2^ and Eu-Tp^CF3^, respectively (Table S4). The crystal structure of the solid powder obtained by sublimation of Eu-Bp^Me^ was also determined (Figure S9) to further confirm that it is Eu-Tp^Me^. No similar phenomenon has been reported in previously reported complexes with pyrazolylborate ligands.

**Figure 4:**
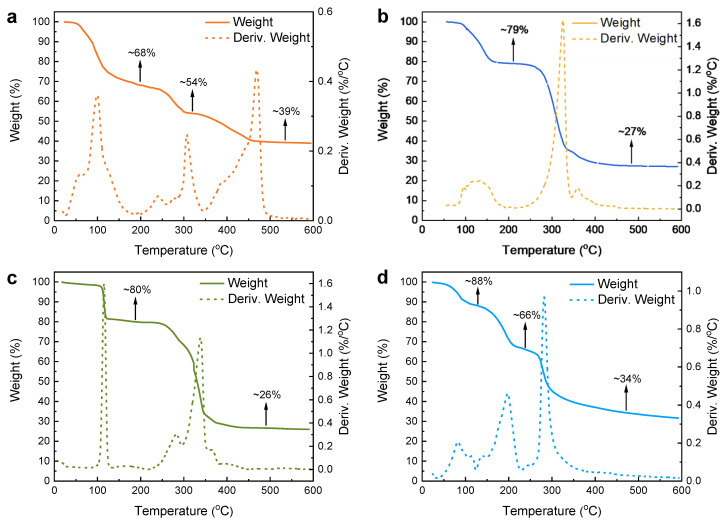
Thermogravimetric curves and derivative thermogravimetric curves of the four Eu(II) complexes: (**a**) Eu-Bp; (**b**) Eu-Bp^Me^; (**c**) Eu-Bp^Me2^; (**d**) Eu-Bp^CF3^.

**Scheme 2:**
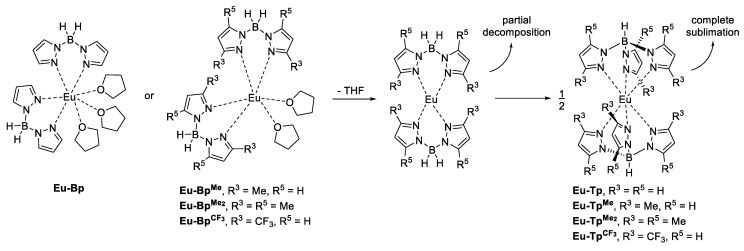
Possible reaction of the four Eu(II) complexes when heated under vacuum or nitrogen atmosphere.

Then, the thermogravimetric analysis (TGA) curves and the derivative thermogravimetric analysis (DTG) curves of the four Eu(II) complexes were measured under nitrogen atmosphere. Based on the TGA data shown in Figure 4 and the calculations shown in Table 3, the possible reaction of these four complexes when heated under vacuum or nitrogen atmosphere is proposed in Scheme 2. Eu-Bp and Eu-Bp^CF3^ have roughly three stages of weight loss in TGA curves. The first step is to lose tetrahydrofuran. According to the calculation shown in Table 3, the theoretical residual mass of Eu-Bp and Eu-Bp^CF3^ after losing tetrahydrofuran is 67% and 88%, respectively, which is consistent with the experimental results (68% and 88% shown in Figure 4a,d, respectively). Then, the residuals are likely to transform to more thermal stable Eu-Tp and Eu-Tp^CF3^, and which are sublimated away. Considering the final residual mass (39% and 34% shown in Figure 4a,d, respectively) is higher than that in theory (24% and 28% shown in Table 3, respectively), the transformation may not complete. Differently, Eu-Bp^Me^ and Eu-Bp^Me2^ have roughly two stages of weight loss. The first step is also to lose tetrahydrofuran coordinated with Eu(II). The experimental results are 79% and 80% (Figure 4b,c, respectively), which are consistent with the theoretical residual mass of 78% and 80% shown in Table 3, respectively. The second step is also proposed that the residuals are transformed to Eu(II) complexes with corresponding Tp ligands and sublimated away. Then, it can be inferred from the residual mass that about half of Eu(II) were formed Eu-Tp^Me^ and Eu-Tp^Me2^. In this way, the theoretical residual mass is both about 26%, which is in good agreement with the experimental results (27% and 26% shown in Figure 4b,c, respectively).

## 3. Materials and Methods

### 3.1. General Methods

^1^H NMR spectra were recorded on 400MHz NMR spectrometer (Bruker, Billerica, MA, USA). Elemental analyses were conducted on a Vario EL cube (GmbH, Hanau, Germany). The syntheses of the complexes were carried out in the glove box. The solvents used in the syntheses and measurements were distilled. Tetrahydrofuran was distilled using Na, NaH and benzophenone under nitrogen atmosphere, n-hexane was distilled using Na and NaH under nitrogen atmosphere. The syntheses of KBp, KBp^Me^, KBp^Me2^ and KBp^CF3^ were according to the literatures [44,45,46].

### 3.2. Synthetic Procedures 

Synthesis of KBp: 0.54 g (10 mmol) potassium borohydride and 1.50 g (22 mmol) pyrazole were added into a 100 mL round-bottom flask, and the mixture was heated to 120 °C under the protection of nitrogen. The hydrogen generated during the reaction was collected by draining water. About 500 mL gas was collected after 3 h. The mixture was cooled to room temperature and washed with toluene. After filtering under reduced pressure and drying naturally, a white solid (1.45 g, 7.8 mmol) was obtained with a yield of 78%. ^1^H NMR (400 MHz, d-DMSO): δ 7.33 (d, *J* = 1.5 Hz, 2H), 7.22 (d, *J* = 1.0 Hz, 2H), 5.93 (t, *J* = 1.7 Hz, 2H), 3.67 (br, 2H), which matches reported literature [48].

Synthesis of KBp^Me^: Similar to that of KBp, instead of 0.54 g (10 mmol) potassium borohydride and 1.80 g (22 mmol) 3-methylpyrazole were used. A white solid (1.60 g, 7.5 mmol) was obtained with a yield of 75%. ^1^H NMR (400 MHz, d-DMSO): δ 7.20 (d, *J* = 1.6 Hz, 2H), 5.68 (d, *J* = 1.5 Hz, 2H), 3.23 (br, 2H), 2.08 (s, 6H), which matches reported literature [48].

Synthesis of KBp^Me2^: Similar to that of KBp, instead of 0.54 g (10 mmol) potassium borohydride and 2.11 g (22 mmol) 3, 5-dimethylpyrazole were used, and the reaction temperature was 160 °C. The mixture was recrystallized with 20 mL mixed solvent (1:3 *v/v* dichloromethane: n-hexane). A white solid (1.94 g, 8.0 mmol) was obtained with a yield of 80%. ^1^H NMR (400 MHz, d-DMSO): δ 5.46 (s, 2H), 3.24 (br, 2H), 2.17 (s, 6H), 2.02 (s, 6H), which matches reported literature [48].

Synthesis of KBp^CF3^: Similar to that of KBp, instead of 0.54 g (10 mmol) potassium borohydride and 2.99 g (22 mmol) 3-trifluoromethylpyrazole were used, and the reaction temperature was 100 °C. The mixture was recrystallized with 20 mL mixed solvent (1:3 *v/v* dichloromethane: n-hexane). A white solid (1.28 g, 4.0 mmol) was obtained with a yield of 40%. ^1^H NMR (400 MHz, d-DMSO): δ 7.52 (d, *J* = 1.5 Hz, 2H), 6.36 (d, *J* = 1.4 Hz, 2H), 3.50 (br, 2H), which matches reported literature [46].

Synthesis of Eu-Bp: 0.41 g (1 mmol) europium(II) iodide and 0.37 g (2 mmol) KBp were dissolved in 10 mL distilled tetrahydrofuran, respectively, in two 100 mL round-bottom flasks. The solution of europium(II) iodide was dropped into the solution of KBp, and the mixture was stirred overnight. Insoluble solid was removed by filtration under reduced pressure. Tetrahydrofuran in the filtrate was removed under reduced pressure. The solid was recrystallized with 10 mL mixed solvent (1:4 *v/v* tetrahydrofuran: n-hexane). After filtering under reduced pressure and drying naturally, a yellow-green solid (0.43 g, 0.65 mmol) was obtained with a yield of 65%. Anal. calcd. for C_12_H_16_B_2_EuN_8_·3C_4_H_8_O: C 43.53%; N 16.92%; H 5.09%; found: C 43.71%; N 16.64%; H 5.38%.

Synthesis of Eu-Bp^Me^: Similar to that of Eu-Bp, instead of 0.41 g (1 mmol) europium(II) iodide and 0.43 g (2 mmol) KBp^Me^ were used. A yellow solid (0.39 g, 0.60 mmol) was obtained with a yield of 60%. Anal. calcd. for C_16_H_24_B_2_EuN_8_·2C_4_H_8_O: C 44.61%; N 17.34%; H 6.24%; found: C 44.65%; N 17.56%; H 6.17%.

Synthesis of Eu-Bp^Me2^: Similar to that of Eu-Bp, instead of 0.41 g (1 mmol) europium(II) iodide and 0.48 g (2 mmol) KBp^Me2^ were used. A green solid (0.39 g, 0.56 mmol) was obtained with a yield of 56%. Anal. calcd. for C_20_H_32_B_2_EuN_8_·2C_4_H_8_O: C 47.88%; N 15.95%; H 6.89%; found: C 47.57%; N 15.72%; H 6.91%.

Synthesis of Eu-Bp^CF3^: Similar to that of Eu-Bp, instead of 0.41 g (1 mmol) europium(II) iodide and 0.64 g (2 mmol) KBp^CF3^ were used, and the mixture was recrystallized from n-hexane. A light green solid (0.30 g, 0.35 mmol) was obtained with a yield of 35%. Anal. calcd. for C_16_H_12_B_2_EuF_12_N_8_·2C_4_H_8_O: C 33.44%; N 13.00%; H 3.27%; found: C 33.34%; N 13.28%; H 3.25%.

### 3.3. Photophysical Measurements

UV-visible absorption spectra were measured by a Shimadzu UV-3600Plus UV-VIS-NIR spectrometer. Excitation and emission spectra were recorded on Edinburgh FLS980 fluorescence spectrophotometer. Luminescence lifetimes were obtained on a single photon counting spectrometer from Edinburgh FLS980 with laser lamp as the excitation source. The data were analyzed by tail fit of the decay profile using a software package provided by Edinburgh Instruments. Absolute photoluminescence quantum yields (PLQYs) were measured using Hamamatsu C9920-02 photoluminescence quantum yield measurement system with integrating sphere. 

The photophysical properties of the complexes were all measured in nitrogen atmosphere. Specifically, the solution of the complexes was prepared and sealed into quartz cells in a glove box for measuring spectra, exited state lifetimes and PLQYs. The solid powder of the complexes was sandwiched between two quartz slices and sealed the edges of the quartz slices with paraffin in a glove box for measuring spectra and excited state lifetimes. The integrating sphere was transferred into a glove box, and the solid powder of the complexes was put into matching quartz cells for measuring PLQYs. All measurements were performed at room temperature.

### 3.4. Thermal Stability Measurements

Thermogravimetric analysis (TGA) and derivative thermogravimetric analysis (DTG) was carried out on Q600SDT instruments at an elevation temperature rate of 10 °C min^−1^ under 100 mL min^−1^ nitrogen flow.

### 3.5. Single Crystal Structure Measurements

The single crystal X-ray diffraction (XRD) data were collected on a Rigaku Mercury CCD diffractometer by using the CrystalClear software. The radiation used in the XRD analysis is the graphite-mon chromated Mo Kα emission line (λ = 0.71069 Å). Structural refinements were conducted with SHELXL-97 or SHELXL-2013 software.

### 3.6. Density Functional Theory (DFT) Calculations

All calculations were performed with the Gaussian 16, revision C.01 program package. The hybrid PBE0 density functional was used for ground state geometry optimizations [49]. MWB53 pseudopotential was used for Eu atom (53 core electrons), and the 6-31G** basis sets were assigned for the other atoms of the complex [50,51]. We had included the atom-pairwise dispersion correction with Becke-Johnson damping (D3BJ) to account for the van der Waals interaction [52,53]. The lowest 50 excited states were estimated by TD-DFT with the hybrid PBE0 density function and optimized ground state geometry. The MWB28 pseudopotential was used for Eu atom (28 core electrons) and def2TZVP basis sets were assigned for the rest atoms. The hole-electron analysis of excited states was performed by Multiwfn software [54,55].

## 4. Conclusions

In summary, four rare earth Eu(II) complexes with bis(pyrazolyl)borate ligands were designed and synthesized, and their structures, photophysical properties and thermal stabilities were studied. Since the steric hindrance of these ligands is relatively small, solvent molecules (tetrahydrofuran) are participated in coordination to form saturated structures. The emission colors of the complexes can be adjusted from blue-green to yellow, indicating that the coordination environments of the complexes can be changed by modifying the ligands, so as to adjust the emission spectra of the complexes. Eu-Bp^Me^ and Eu-Bp^Me2^ show high PLQYs both as solid powder (100%) or in tetrahydrofuran solution (1 × 10^−3^ M) (>75%). However, when there are too many solvent molecules involved in coordination, or they are too close to central Eu(II), the luminescence of the complexes may be quenched. As a result, Eu-Bp and Eu-Bp^CF3^ show much lower PLQYs as solid powder (<40%) or in tetrahydrofuran solution (1 × 10^−3^ M) (<15%). In addition, when heated under vacuum or nitrogen atmosphere, these complexes can finally transform into more thermal stable complexes of Eu(II) and corresponding Tp ligands. These new Eu(II) complexes can provide reference for the study of the relationship between luminescence and structure, and their interesting transformation when heated may also be helpful to the study of thermal stability. Their high PLQYs and short excited state lifetimes make them have potential applications in organic light emitting diodes. However, there are still some problems to overcome, such as the poor thermal stability and air stability.

## Figures and Tables

**Table 1:** The Eu-N and Eu-O bond lengths of the four Eu(II) complexes. The labels for coordinated nitrogen atoms and oxygen atoms are shown in Figure 2.

Bond	Eu-Bp (Å)	Eu-Bp^Me^ (Å)	Eu-Bp^Me2^ (Å)	Eu-Bp^CF3^ (Å)
Eu-N1	2.638	2.606	2.630	2.665
Eu-N2	2.654	2.664	2.651	2.706
Eu-N3	2.642	2.619	2.630	2.681
Eu-N4	2.674	2.648	2.651	2.716
Eu-O1	2.603	2.571	2.579	2.558
Eu-O2	2.618	2.572	2.579	2.558
Eu-O3	2.666	/	/	/

**Table 2:** Photophysical data of the four Eu(II) complexes in tetrahydrofuran solution (1 × 10^−3^ M) and as solid powder.

Complex	State	λ_em_ (nm)	PLQYs (%)	τ (ns)	FWHMs (nm)	CIE	*k*_r_ (10^6^ s^−1^)	*k*_nr_ (10^6^ s^−1^)
Eu-Bp	solution	556	11 ^a^	78	96	(0.41, 0.55)	1.41	11.41
	solid	542	37 ^b^	379	94	(0.38, 0.58)	0.98	1.67
Eu-Bp^Me^	solution	546	76 ^a^	530	88	(0.39, 0.57)	1.89	0.60
	solid	554	100 ^c^	543	86	(0.42, 0.56)	1.84	0
Eu-Bp^Me2^	solution	553	76 ^a^	475	83	(0.40, 0.56)	1.60	0.51
	solid	526	100 ^c^	488	66	(0.28, 0.63)	2.05	0
Eu-Bp^CF3^	solution	541	5 ^a^	31	109	(0.36, 0.54)	1.61	30.59
	solid	497	20 ^d^	153	81	(0.22, 0.44)	1.31	5.24

^a^ λ_ex_ = 280 nm, ^b^ λ_ex_ = 320 nm, ^c^ λ_ex_ = 400 nm, ^d^ λ_ex_ = 300 nm, where λ_ex_ represents excitation wavelength.

**Table 3:** Theoretical calculation of thermogravimetric residual mass of the four Eu(II) complexes.

Complex		Initial Value	First Weight Loss (-THF)	Second Weight Loss (-Eu-Tp ^a^)
Eu-Bp	Molecular formula	C_24_H_40_B_2_EuN_8_O_3_	C_12_H_16_B_2_EuN_8_	1/2 C_6_H_12_B_2_EuN_4_
	Molecular weight	666.3	447.1	157.5
	Residual mass	100%	67%	24%
Eu-Bp^Me^	Molecular formula	C_24_H_40_B_2_EuN_8_O_2_	C_16_H_24_B_2_EuN_8_	1/2 C_8_H_16_B_2_EuN_4_
	Molecular weight	647.3	503.2	171.5
	Residual mass	100%	78%	26%
Eu-Bp^Me2^	Molecular formula	C_28_H_48_B_2_EuN_8_O_2_	C_20_H_32_B_2_EuN_8_	1/2 C_10_H_20_B_2_EuN_4_
	Molecular weight	703.3	559.2	185.6
	Residual mass	100%	80%	26%
Eu-Bp^CF3^	Molecular formula	C_24_H_28_B_2_EuF_12_N_8_O_2_	C_16_H_12_B_2_EuF_12_N_8_	1/2 C_8_H_8_B_2_EuF_8_N_4_
	Molecular weight	863.2	763.2	243.5
	Residual mass	100%	88%	28%

^a^ It represents Eu-Tp, Eu-Tp^Me^, Eu-Tp^Me2^ and Eu-Tp^CF3^, respectively.

## Data Availability

CCDC 2167575, 2167572-2167574 contain the supplementary crystallographic data for this paper. These data can be obtained free of charge via www.ccdc.cam.ac.uk/data_request/cif, or by emailing data_request@ccdc.cam.ac.uk, or by contacting The Cambridge Crystallographic Data Centre, 12 Union Road, Cambridge CB2 1EZ, UK; fax: +44-1223-336033.

## Supplementary Materials

The following are available online at https://www.mdpi.com/article/10.3390/molecules27228053/s1, Table S1: Crystallographic data for Eu-Bp, Eu-Bp^Me^, Eu-Bp^Me2^ and Eu-Bp^CF3^, Figure S1: UV-Vis absorption spectra of the four Eu(II) complexes in tetrahydrofuran solution (1 × 10^−3^ M), Table S2: UV-Vis absorption and excitation data of the four Eu(II) complexes in tetrahydrofuran solution (1 × 10^−3^ M), Figure S2: Excitation spectra of the four Eu(II) complexes, Figure S3: CIE 1931 chromaticity diagrams of the four Eu(II) complexes, Figure S4: Experimental and TD-DFT calculated absorption spectra of the four Eu(II) complexes, Table S3: Calculated lowest excited states energies (*E*), wavelengths (*λ*) and oscillator strengths (*f*) of the four Eu(II) complexes, Figure S5: Hole-electron analysis of Eu-Bp, Figure S6: Hole-electron analysis of Eu-Bp^Me^, Figure S7: Hole-electron analysis of Eu-Bp^Me2^, Figure S8: Hole-electron analysis of Eu-Bp^CF3^,Table S4: Changes of luminescent colors and elemental analyses of the Eu(II) complexes before and after sublimation, Figure S9: The crystal structure of Eu-Tp^Me^.

## Sample Availability

Samples of the compounds KBp, KBp^Me^, KBp^Me2^, KBp^CF3^, Eu-Bp, Eu-Bp^Me^, Eu-Bp^Me2^ and Eu-Bp^CF3^ are available from the authors.
