# Supplementary material for: Rare Earth Complexes of Europium(II) and Substituted Bis(pyrazolyl)borates with High Photoluminescence Efficiency
Source: Molecules. 2022 Nov 20;27(22):8053. doi: 10.3390/molecules27228053 (PMC9694868; doi:10.3390/molecules27228053)
Supplement: Supplementary file 1 [file molecules-27-08053-s001.zip › molecules-2006796-supplementary.pdf]

Supplementary Materials

Rare Earth Complexes of Europium(II) and Substituted Bis(pyrazolyl)borates with High Photoluminescence Efficiency

Ruoyao Guo [1], Zifeng Zhao [1], Aoben Wu [1], Yuqin Li [2], Kezhi Wang [2], Zuqiang Bian [1] and Zhiwei Liu [1,*]

[1] Beijing National Laboratory for Molecular Sciences, State Key Laboratory of Rare Earth Materials Chemistry and Applications, College of Chemistry and Molecular Engineering, Peking University, Beijing 100871, China
[2] Beijing Key Laboratory of Energy Conversion and Storage Materials, College of Chemistry, Beijing Normal University, Beijing 100875, China
* Correspondence: zwliu@pku.edu.cn

Table S1. Crystallographic data for Eu-Bp, Eu-Bp^{Me}, Eu-Bp^{Me2} and Eu-Bp^{CF3}.

Complex	Eu-Bp	Eu-Bp^{Me}	Eu-Bp^{Me2}	Eu-Bp^{CF3}
chemical formula	$C_{24}H_{40}B_2EuN_8O_3$	$C_{24}H_{40}B_2EuN_8O_2$	$C_{28}H_{48}B_2EuN_8O_2$	$C_{24}H_{28}B_2EuF_{12}N_8O_2$
formula weight	662.22	646.22	702.32	862.12
crystal size (mm)	0.18×0.05×0.04	0.08×0.06×0.03	0.25×0.22×0.18	0.22×0.05×0.04
temperature (K)	180	180	180	180
crystal system	triclinic	monoclinic	monoclinic	monoclinic
space group	P -1	P 21/n	C 2/c	P 21/n
a(Å)	9.1027(4)	12.2995(3)	18.4999(3)	10.9107(2)
b(Å)	10.2412(5)	13.9414(4)	8.3478(1)	18.6778(3)
c(Å)	17.3738(3)	17.5154(5)	21.6822(4)	16.0633(2)
α(°)	94.388(3)	90	90	90
β(°)	96.063(3)	90.486(2)	90.147(2)	90.768(1)
γ(°)	109.865(4)	90	90	90
V(Å^3)	1503.75(11)	3003.30(14)	3348.45(9)	3273.21(9)
Z	2	4	4	4
$\rho_{(calc)}$ (g/cm^3)	1.463	1.429	1.393	1.749
F (000)	674.0	1316.0	1444.0	1700.0

θ range (deg)	2.311-26.370	2.489-25.027	2.677-30.658	2.243-29.532
GOF	1.050	1.048	1.029	1.004
R_1/wR_2 [$I>2\sigma(I)$]	0.0488/0.1160	0.0295/0.0684	0.0204/0.0523	0.0252/0.0635
R_1/wR_2 (all data)	0.0544/0.1199	0.0385/0.0721	0.0215/0.0529	0.0338/0.0670

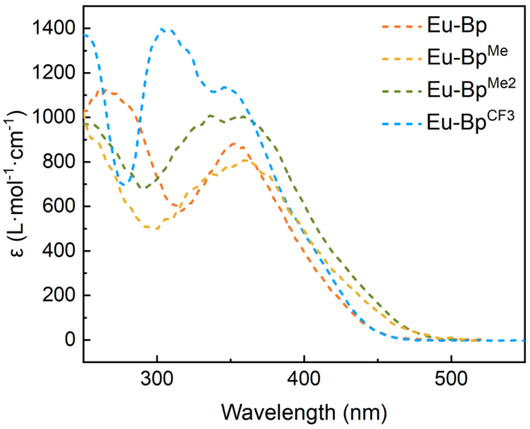

Figure S1. UV-Vis absorption spectra of the four Eu(II) complexes in tetrahydrofuran solution (1×10^{-3} M).

Table S2. UV-Vis absorption and excitation data of the four Eu(II) complexes in tetrahydrofuran solution (1×10^{-3} M).

Complex	Absorption peaks (nm)	ϵ (L mol^{-1} cm^{-1})	Excitation peaks (nm)
Eu-Bp	265, 353	1123, 882	290, 369
Eu-Bp^{Me}	259, 360	897, 806	272, 327, 364
Eu-Bp^{Me2}	256, 336, 354	966, 1008, 1000	285, 391
Eu-Bp^{CF3}	252, 303, 346	1365, 1397, 1134	282, 343, 366

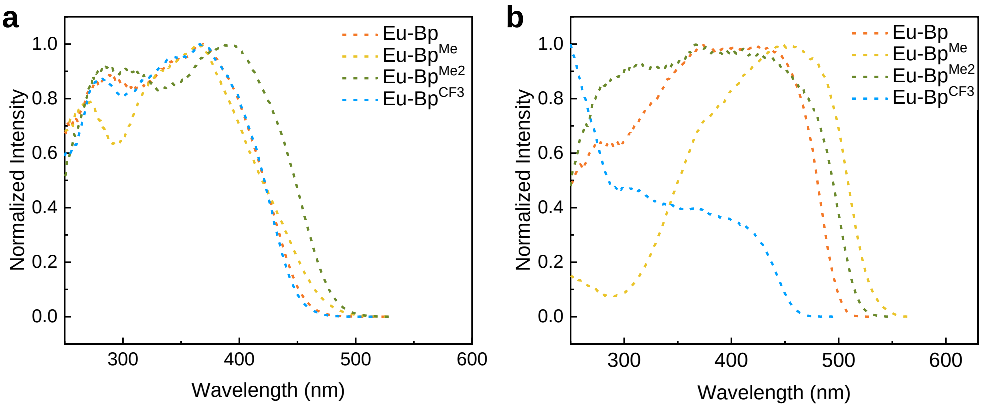

Figure S2. Excitation spectra of the four Eu(II) complexes. (a) Excitation spectra in tetrahydrofuran solution (1×10^{-3} M). The emission wavelengths are 550 nm, 550 nm, 550 nm and 560 nm for Eu-Bp, Eu-Bp^{Me}, Eu-Bp^{Me2} and Eu-Bp^{CF3}, respectively. (b) Excitation spectra as solid powder. The emission wavelengths are 560 nm, 580 nm, 560 nm and 500 nm for Eu-Bp, Eu-Bp^{Me}, Eu-Bp^{Me2} and Eu-Bp^{CF3}, respectively.

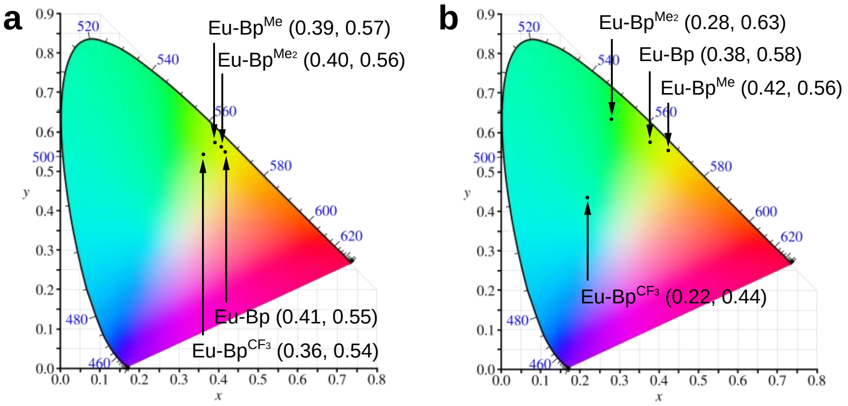

Figure S3. CIE 1931 chromaticity diagrams of the four Eu(II) complexes. (a) CIE 1931 chromaticity diagram in tetrahydrofuran solution (1×10^{-3} M). (b) CIE 1931 chromaticity diagram as solid powder.

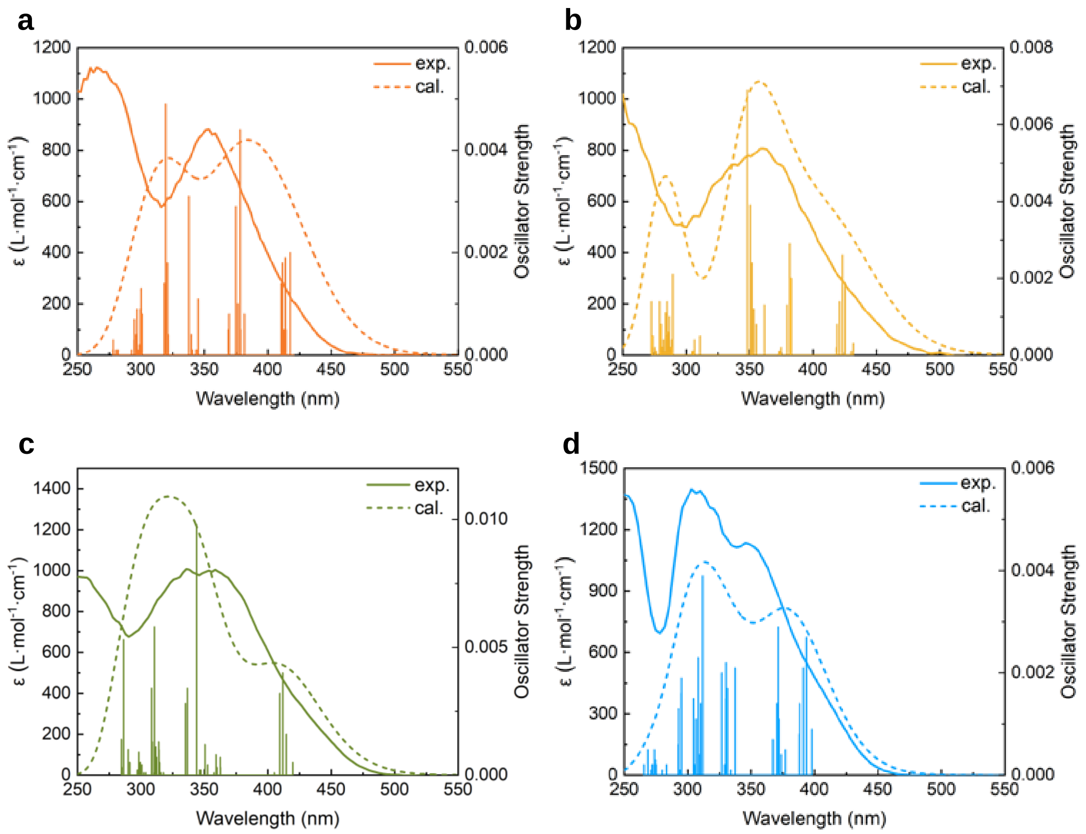

Figure S4. Experimental (solid line) and TD-DFT calculated (dashed line) absorption spectra of the four Eu(II) complexes. (a) Eu-Bp. (b) Eu-Bp^{Me}. (c) Eu-Bp^{Me2}. (d) Eu-Bp^{CF3}. Oscillator strengths for the electronic transitions are shown as vertical lines.

Table S3. Calculated lowest excited states energies (*E*), wavelengths (*λ*) and oscillator strengths (*f*) of the four Eu(II) complexes. Experimental values are given in parentheses.

**Complex**	Excited state	***E*** **(eV)**	***λ*** **(nm)**	*f*
**Eu-Bp**	S_2[a]	2.97 (3.51)	418 (353)	0.0020
**Eu-Bp^{Me}**	S_1	2.87 (3.44)	432 (360)	0.0003
**Eu-Bp^{Me2}**	S_1	2.95 (3.50)	420 (354)	0.0005
**Eu-Bp^{CF3}**	S_1	3.12 (3.58)	398 (346)	0.0009

[a] The oscillator strength of S_1 is 0.

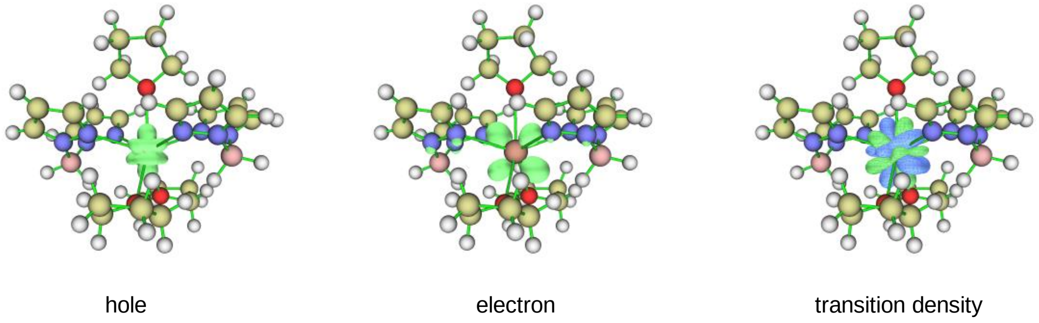

Figure S5. Hole-electron analysis of Eu-Bp with isosurface value 0.005.

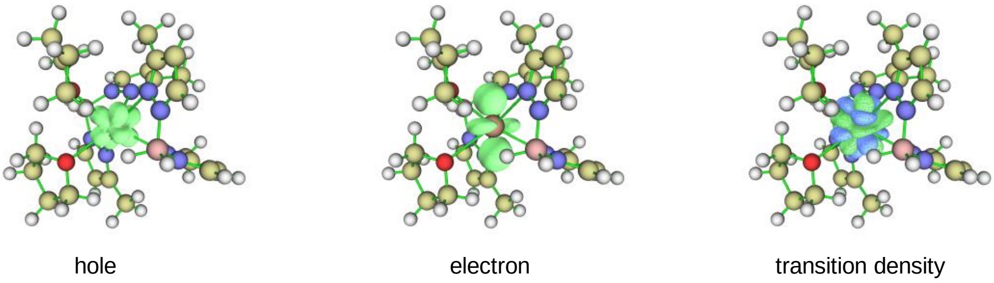

Figure S6. Hole-electron analysis of Eu-Bp^{Me} with isosurface value 0.005.

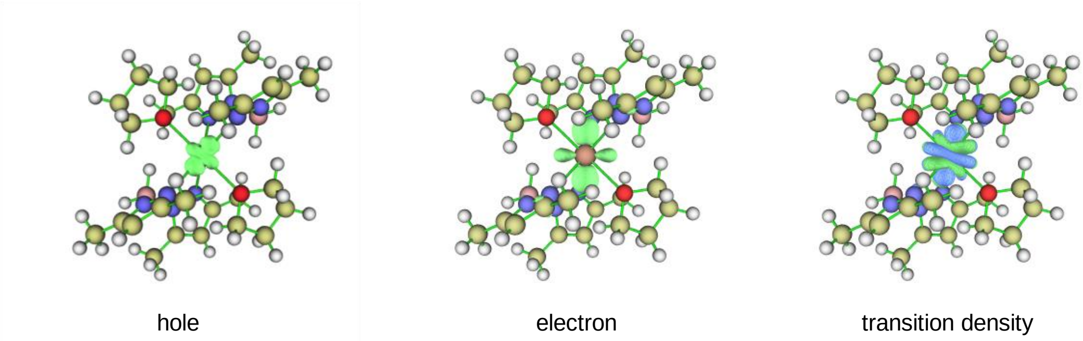

Figure S7. Hole-electron analysis of Eu-Bp^{Me2} with isosurface value 0.005.

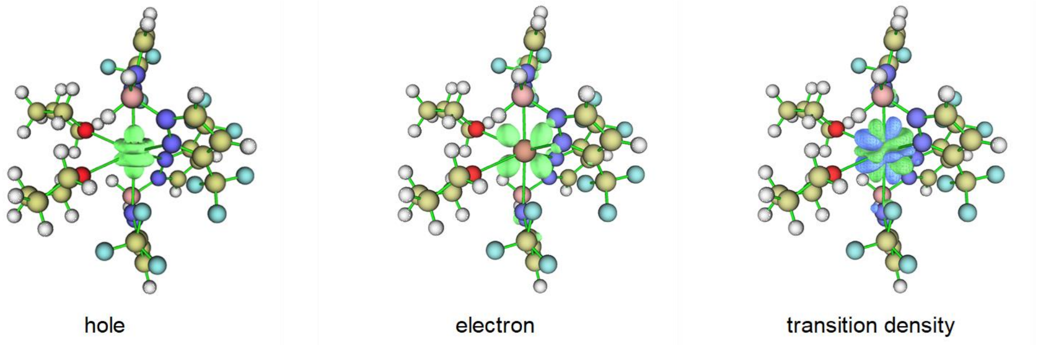

Figure S8. Hole-electron analysis of Eu-Bp^{CF3} with isosurface value 0.005.

Table S4. Changes of luminescent colors and elemental analyses of the Eu(II) complexes before and after sublimation.

Complex	Luminescent color		Elemental analysis C (%)	H (%)	N (%)
Eu-Bp (before sublimation)	yellow-green	calcd.	41.62	5.67	18.32
		found	41.71	5.68	18.34
Eu-Tp (after sublimation)	red	calcd.	37.40	3.49	29.08
		found	37.43	3.70	29.15
Eu-Bp^{Me} (before sublimation)	yellow	calcd.	43.84	6.07	17.94
		found	43.65	6.17	17.86
Eu-Tp^{Me} (after sublimation)	orange	calcd.	43.53	4.87	25.38
		found	43.67	4.93	25.48
Eu-Bp^{Me2} (before sublimation)	green	calcd.	47.88	6.89	15.95
		found	47.57	6.91	15.72
Eu-Tp^{Me2} (after sublimation)	orange	calcd.	48.28	5.94	22.52
		found	48.49	6.13	22.59
Eu-Bp^{CF3} (before sublimation)	blue-green	calcd.	33.44	3.27	13.00
		found	33.34	3.25	13.28
Eu-Tp^{CF3} (after sublimation)	blue	calcd.	29.24	1.43	17.05
		found	29.09	1.24	17.19

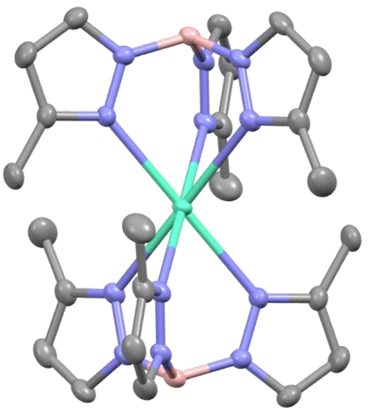

Figure S9. The crystal structure of Eu-Tp^{Me}. For clarity, the hydrogen atoms are omitted. Eu atoms are represented in cyan, O in red, B in pink, N in blue and C in gray.
